# Effects of dietary niacin supplementation on performance, egg quality and yolk antioxidant status in laying quails

**DOI:** 10.1007/s11250-025-04794-w

**Published:** 2025-12-09

**Authors:** Esra Tuğçe Gül, Osman Olgun, Gözde Kılınç, Alpönder Yıldız, Ainhoa Sarmiento-García

**Affiliations:** 1 Department of Animal Science, Agriculture Faculty, Selcuk University, Konya, 42130 Türkiye; 2 Department of Food Processing, Suluova Vocational Schools, Amasya University, Amasya, 05500 Türkiye; 3 Departamento de Producción Animal, Facultad de Veterinaria, Universidad de León, León, 24007 España

**Keywords:** Antioxidant capacity, Egg quality, Niacin, Performance, Quail

## Abstract

This study evaluated the effects of graded dietary niacin supplementation on performance, egg quality, and yolk antioxidant capacity in laying quails. A total of 180 birds were assigned to six treatments (0, 10, 20, 40, 80, and 120 mg/kg niacin) for 12 weeks under a completely randomised design. The control diet contained 25.32 mg/kg niacin. Niacin supplementation produced modest but statistically significant effects on certain productive performance (*P* < 0.05). Egg production and egg mass improved up to 40 mg/kg, showing linear trends (*P* = 0.034 and *P* = 0.022, respectively), while feed conversion ratio was most efficient at the same level (*P* = 0.040). Supplementation with 10 mg/kg enhanced shell strength (*P* = 0.034) and thickness (*P* = 0.030), indicating a dose-specific response at low inclusion. Internal egg quality (albumen index and Haugh unit) increased linearly (*P* ≤ 0.001), reaching their highest values at 120 mg/kg. Yolk antioxidant capacity (DPPH) rose by 26.8%, whereas lipid peroxidation (MDA) declined by 55% at 120 mg/kg (both *P* < 0.05). Overall, niacin influenced productivity and antioxidant status in a dose-dependent but non-linear manner, suggesting distinct optimal levels for performance (40 mg/kg) and oxidative protection (120 mg/kg). Further long-term and large-scale studies are warranted before revising niacin recommendations for laying quails.

## Introduction

Vitamins are essential micronutrients required for a wide range of physiological processes, including metabolic regulation, oxidative balance, and immune function in poultry (Akinyemi and Adewole 2021). In recent years, the strategic use of vitamins has gained renewed importance as part of nutritional approaches aimed at maintaining productivity and health while reducing antibiotic reliance in animal production systems (Ahmadian et al. 2021; Akinyemi and Adewole 2021). However, most existing vitamin requirements are based on outdated NRC (1994) standards that may not reflect the metabolic demands of modern poultry genotypes or non-conventional species such as quail, where nutritional data remain scarce (Olgun et al. 2022).

Niacin, also known as vitamin B_3_, comprises two biologically active forms: nicotinic acid (pyridine-3-carboxylic acid) and nicotinamide (pyridine-3-carboxamide). which serve as precursors for the essential coenzymes NAD⁺ and NADP⁺. These coenzymes are integral to glycolysis, oxidative phosphorylation, and lipid metabolism and also act as redox mediators involved in free-radical scavenging and oxidative protection (Ahmadian et al. 2021). Although nicotinic acid and nicotinamide are metabolically equivalent, they differ in their pharmacological properties. Nicotinic acid has been shown to modulate lipid metabolism by reducing the hepatic synthesis of triglycerides, LDL-C, and VLDL-C, and by increasing HDL-C levels through the downregulation of diacylglycerol acyltransferase 2 (DGAT2) expression (Less and Galili 2008; Adebowale et al. 2019).

Niacin supplementation has shown promising effects in poultry nutrition, particularly in enhancing growth performance, improving feed efficiency, and modulating lipid metabolism. Positive outcomes have been reported, including increased body weight gain (BWG), reduced abdominal fat deposition, enhanced meat quality, and improved serum lipid profiles (El-Husseiny et al. 2008; Adebowale et al. 2018; Baghban-Kanani et al. 2019). In laying hens, niacin has also been associated with improved egg production (EP), better feed conversion, and enhanced eggshell quality (Gungor et al. 2003; El-Husseiny et al. 2008; Elsherif et al. 2019; Baghban-Kanani et al. 2019). These benefits are mainly attributed to its role in energy metabolism and oxidative stress regulation.

However, most studies have focused on broilers or laying hens, while evidence in laying quails remains limited and sometimes inconsistent. Previous research has mainly addressed productive or egg-quality responses, with only a few reports examining niacin’s antioxidant effects. In those cases, systemic oxidative markers have often shown negligible or variable responses (Baghban-Kanani et al. 2019), leaving the specific role of niacin in yolk oxidative stability and its potential interaction with egg quality largely unresolved.

The Japanese quail (*Coturnix coturnix japonica*) is a species of growing economic relevance due to its rapid growth, early sexual maturity, and increasing consumer demand for eggs and meat (Vargas-Sánchez et al. 2019; Sarmiento-García et al. 2023a). Yet, the niacin requirement for optimal productivity and antioxidant protection in this species has not been well defined. The current NRC (1994) recommendation of 20 mg/kg is likely insufficient under modern conditions, and higher levels may be required to sustain performance and egg quality.

Therefore, the current research was designed to evaluate the effects of graded dietary niacin supplementation on performance, egg quality, and yolk antioxidant capacity in laying quails. It was hypothesised that niacin would differentially influence these traits depending on the inclusion level, with moderate supplementation optimising productive performance and shell quality, and higher levels enhancing yolk antioxidant protection.

## Materials and methods

### Experimental design and animal management

The experiment was carried out at an indoor experimental facility located in Selçuklu, Konya, Türkiye (38°01′36″N, 32°30′45″E). A total of 180 healthy Japanese laying quails (*Coturnix coturnix Japonica*), 11 weeks of age and with a mean body weight (BW) of 256.2 ± 13.3 g, were used. The 12-week feeding period was selected to represent a complete production phase in laying quails, covering the early to mid-laying cycle during which egg quality and antioxidant status are most stable and nutritionally responsive. The sample size was determined based on previous comparable trials (Olgun et al. 2022; Sarmiento-García et al. 2023a), which demonstrated adequate statistical power (β > 0.8) for detecting treatment differences of ≥ 5% in productive traits.

Birds were randomly allotted to six dietary treatments (0, 10, 20, 40, 80, and 120 mg/kg niacin) with six replicates of five quails per replicate (*n* = 30 per treatment) following a completely randomised design. Quails were kept in sanitised wire-mesh cages (30 × 45 cm; area = 1350 cm² per cage), corresponding to a stocking density of 270 cm² per bird, which falls within the recommended range for laying quails under controlled environmental conditions. The facility was mechanically ventilated, and the ambient temperature was maintained at 20 ± 2 °C with a relative humidity of 60 ± 5%., as monitored daily using a digital thermo-hygrometer. A 16 h light: 8 h dark photoperiod (15 lx at cage level) was provided. Feed and water were available ad libitum, and cages were cleaned weekly.

The health status of the quails was monitored daily by a licensed veterinarian. Birds followed the routine vaccination schedule recommended for small-scale quail production in Türkiye, including Newcastle disease (LaSota strain, day 10 and booster at week 8) and avian influenza (H5 inactivated vaccine, week 12). No antibiotic or antiparasitic medication was administered during the trial. Mortalities and abnormal behaviours were recorded, but no clinical signs of disease occurred during the experiment.

The dietary treatments were formulated by supplementing a basal mash diet, using a commercial-grade niacin product (Jiangxi Brother Pharmaceutical Co., Ltd., Jiujiang, China). Niacin was provided as nicotinic acid, the biologically active form commonly used in poultry supplementation. The basal diet contained 25.32 mg/kg of naturally occurring niacin, as determined by chemical analysis. Therefore, the “0 mg/kg” treatment represented a non-supplemented control rather than a niacin-deficient diet. All other treatments were formulated by supplementing this basal diet with additional niacin to reach total dietary levels of approximately 35, 45, 65, 105, and 145 mg/kg. This baseline concentration was considered when interpreting dose–response relationships, which thus reflect the effect of supplemental rather than total niacin intake. The selected range was based on the NRC (1994) recommendation of 20 mg/kg for poultry and was extended in line with previous studies in hens and turkeys reporting benefits at higher levels (El-Husseiny et al. 2008; Adebowale et al. 2019). This design allowed testing of both sub-recommended, recommended, and supra-nutritional doses relevant to current practice.

All diets were formulated to be isoenergetic and isonitrogenous, with ingredient ratios adjusted to ensure nutritional balance across treatments. Diets were mixed thoroughly to achieve homogeneity and provided in mash form. The feeding trial lasted 12 weeks, during which quails were housed under standardised conditions and received feed and water ad libitum. Nutrient formulation followed NRC (1994) guidelines for laying quail.

The proximate composition of the basal diet was determined according to official AOAC methods (2006) and expressed on an as-fed basis. Crude protein was analysed using the Kjeldahl method (AOAC 990.03), and ether extract was quantified by Soxhlet extraction (AOAC 2003.06). Moisture content was measured by oven-drying at 105 °C (AOAC 2001.12), and ash was determined via muffle furnace incineration. Mineral composition (Ca, P, Na, K, Mg, and trace elements) was determined by an accredited external laboratory following validated AOAC procedures. All analyses were performed in duplicate, and results were expressed on an as-fed basis. Detailed ingredient composition and nutrient content of the diets are presented in Table 1.

**Table 1:** Ingredients and nutrient content of the basal diet, as fed

Ingredients	g/kg	Nutrients	g/kg
Corn	537.0	Metabolizable energy, kcal/kg	2900
Soybean meal	351.0	Crude protein^2^	200.1
Soybean oil	37.4	Crude oil^2^	59.15
Limestone	56.0	Crude fibre^2^	27.23
Dicalcium phosphate	11.4	Moisture^2^	129.51
Salt	3.0	Calcium^2^	24.99
Premix^1^	1.0	Total phosphorus^2^	6.42
DL-methionine	2.2	Available phosphorus	3.50
Toxin binder	1.0	Lysine	11.03
		Methionine	4.55
		Methionine + cystine	3.74
		Niacin, mg/kg	25.32

^1^Premix: 13,000 IU vitamin A; 5,000 IU vitamin D_3_; 80 mg vitamin E; 4 mg vitamin K_3_; 5 mg thiamine; 9 mg riboflavin; 25 mg pantothenic acid; 5 mg pyridoxine; 0.35 mg biotin; 2.5 mg folic acid; 0.02 mg vitamin B_12_ mg; 16 mg copper; 1.25 mg; 20 mg iron; 120 mg manganese; 0.30 mg selenium; 120 mg zinc incorporated at 1 g per kg of feed

^2^Analysed value

#### Productive performance measurements

Performance parameters were recorded to evaluate the effects of dietary niacin supplementation. Initial and final BW were measured using a precision scale (± 0.01 g), and BWG was calculated as the difference between final and initial BW (BWG = final BW − initial BW). Daily feed intake (FI; g/bird/day) was determined by subtracting the feed residues from the total amount offered, using the procedure outlined by Olgun et al. (2022). Egg production (r) (%) was expressed as the ratio of the total number of eggs collected daily to the number of quails in each replicate, multiplied by 100. Egg weight (EW; g) was recorded using an electronic scale, and egg mass (EM; g/bird/day) was calculated by multiplying EP by average EW. Feed conversion ratio (FCR) was determined by dividing FI by EM, as described by Sarmiento-García et al. (2024).

### Evaluation of shell integrity and composition

For the evaluation of egg quality, a total of 300 eggs were randomly collected during the final 72 h of the experiment (50 eggs per treatment; 8–9 eggs per replicate). All measurements were performed at the Egg Quality Laboratory of Selçuk University (Konya, Türkiye). External defects, including cracking or breakage, were recorded and expressed as a percentage of the total sample evaluated. Shell resistance to compression was quantified at the blunt pole of the egg using a calibrated force gauge (Egg Force Reader, Orka Food Tech, Israel), which applies pressure until fracture. Shell thickness was measured in microns at three anatomical points (broad, equatorial, and narrow ends) using a digital micrometre (Mitutoyo, 0.01 mm resolution). A mean thickness value was derived for each egg. To calculate the proportional contribution of the shell to total EM, each shell was manually cleaned, left to air-dry, and weighed with precision. The resulting values were expressed as a percentage of individual EW.

### Quantification of yolk lipid peroxidation and antioxidant capacity

The oxidative status of egg yolks was assessed by quantifying malondialdehyde (MDA) concentration and evaluating antioxidant activity via 2,2-diphenyl-1-picrylhydrazyl (DPPH) radical scavenging capacity. A total of 144 freshly collected eggs were randomly selected (24 per treatment; four per replicate). The sample size was determined to allow triplicate laboratory measurements per replicate, ensuring data reliability and compliance with previous methodological standards in similar quail studies (Sarmiento-García et al. 2023a).

Lipid peroxidation was estimated using a thiobarbituric acid reactive substances (TBARS) method, based on the protocols described by Kilic and Richards (2003) and further refined by Sarmiento-García et al. (2023a). Briefly, equal volumes (1 mL) of yolk sample and a trichloroacetic acid (TCA) reagent (containing 7.5% TCA, 0.1% EDTA, and 0.1% propyl gallate) were mixed with 1 mL of 0.02 M thiobarbituric acid (TBA). The mixture was incubated under controlled conditions, and absorbance was measured at 530 nm using a UV-visible spectrophotometer. MDA levels were calculated using a standard calibration curve and expressed as µmol of MDA per kilogram of yolk.

The antioxidant potential of yolk samples was analysed using the DPPH radical scavenging assay, following the general procedure proposed by Sacchetti et al. (2005), with adaptations introduced by Olgun et al. (2022). After incubation with the DPPH reagent, absorbance was recorded at 517 nm. Ethanol (95%) served as the blank. The radical scavenging capacity was expressed as a percentage of inhibition relative to the control sample.

### Statistical analysis

Data were analysed using SPSS software (version 22.0; IBM Corp., Armonk, NY, USA). The Shapiro–Wilk test and Levene’s test were applied to verify the assumptions of normality and homogeneity of variances. When assumptions were met, data were analysed using a mixed model, in which dietary niacin level was included as a fixed effect and cage (replicate) was treated as a random effect to account for the hierarchical structure of the data and avoid pseudo-replication. For performance traits, the cage served as the experimental unit, while individual eggs were the unit for egg quality and antioxidant analyses. Results are presented as mean values accompanied by their respective standard errors (± SEM). Differences among treatment groups were considered statistically significant at *P* < 0.05, and trends were noted when P values ranged between 0.05 and 0.10. The linear and quadratic effects of increasing dietary niacin levels were evaluated using orthogonal polynomial contrasts to assess dose–response relationships.

## Results

### Performance

As shown in Table 2, no significant differences were observed in initial BW among the experimental groups (*P* > 0.05), confirming the uniformity of the birds at the start of the trial. Egg production responded significantly to dietary niacin levels (*P* = 0.034).

**Table 2:** Effect of dietary niacin supplementation on performance parameters of laying quails (*n* = 180)

Parameters	Niacin levels, mg/kg						SEM	Anova	Linear	Quadratic
	0	10	20	40	80	120				
IBW (g)	251.90	260.40	255.40	250.40	261.40	257.50	1.857	0.463	0.434	0.939
FBW (g)	273.50	280.90	276.80	272.70	278.30	275.00	2.332	0.930	0.928	0.923
BWG (g)	21.60	20.50	21.40	22.30	16.90	17.50	1.936	0.958	0.415	0.963
EP (%)	91.83^ab^	90.69^b^	91.83^ab^	92.92^a^	90.53^b^	90.71^b^	0.257	0.034	0.113	0.269
EW (g)	13.22	13.44	13.16	13.11	13.50	12.48	0.111	0.101	0.061	0.113
EM (g/day/quail)	12.14	12.19	12.08	12.18	12.21	11.32	0.104	0.087	0.022	0.064
FI (g/day/quail)	33.54	34.98	33.98	32.97	33.39	33.25	0.225	0.121	0.122	0.499
FCR	2.766	2.871	2.814	2.711	2.744	2.951	0.0281	0.127	0.218	0.040

^a, b^ Means with different superscripts in the same row were significantly different at *P* < 0.05. S.E.M.: Standard Error of the Mean. IBW: Initial Body Weight, FBW: Final Body Weight, BWG: Body Weight Gain, EP: Egg Production, EW: Egg Weight, EM: Egg Mass, FI: Feed Intake, FCR: Feed Conversion Ratio

Birds receiving 40 mg/kg niacin exhibited the highest laying rate (92.92%), which was significantly greater than that of the 10, 80, and 120 mg/kg groups (*P* = 0.034). This value represented a 1.2% increase compared with the control (91.83%), indicating a modest improvement in productivity. A numerical trend towards a linear increase in EP was observed, although it did not reach statistical significance (*P* > 0.05).

Egg weight was not significantly influenced by niacin inclusion (*P* > 0.05), although a slight linear tendency was detected. Similarly, EM (g/day per quail) did not differ significantly among treatments (overall ANOVA *P* = 0.087), but a trend toward a linear increase was detected (linear contrast *P* = 0.022), with higher niacin levels associated with a modest rise in egg output.

Feed intake remained unaffected by the treatments (*P* > 0.05). The FCR showed a significant quadratic response to dietary niacin level (*P* = 0.040), despite no overall difference across treatments (*P* > 0.05). The most efficient FCR was obtained in birds supplemented with 40 mg/kg niacin (2.711), representing a 2.0% improvement compared with the control group (2.766), whereas the least favourable value occurred at 120 mg/kg. This indicates a moderate but measurable enhancement in feed efficiency at the 40 mg/kg inclusion level.

### Eggshell strength and structural parameters

As detailed in Table 3, the percentage of damaged eggs was not significantly affected by dietary niacin supplementation (*P* > 0.05). Mean values ranged from 0.00% to 0.35%, with no clear dose-dependent pattern, and neither linear nor quadratic trends were observed.

**Table 3:** Structural and physical eggshell characteristics of quail eggs (*n* = 300) under different niacin supplementation levels

Parameters	Niacin levels, mg/kg						SEM	Anova	Linear	Quadratic
	0	10	20	40	80	120				
Damaged eggs (%)	0.16	0.32	0.34	0.35	0.35	0.00	0.084	0.810	0.468	0.220
Shell strength (N)	10.11^b^	12.40^a^	11.86^a^	11.37^ab^	11.13^ab^	11.93^a^	0.219	0.034	0.407	0.804
Shell ratio (%)	7.84	8.31	8.17	8.04	8.17	8.33	0.067	0.271	0.174	0.954
Shell thickness (µm)	203.78^b^	221.83^a^	217.11^a^	212.40^ab^	210.30^ab^	216.48^a^	1.698	0.030	0.656	0.929

^a, b^Means with different superscripts in the same row were significantly different at *P* < 0.05. SEM: standard error means

Shell strength was significantly influenced by dietary niacin level (*P* = 0.034). Birds receiving 10, 20, and 120 mg/kg niacin produced eggs with stronger shells than the control group. The highest value was recorded at 10 mg/kg (12.40 N), representing a 22.7% increase compared with the non-supplemented birds (10.11 N). Shell thickness also improved by 8.9% at this level (*P* = 0.030), indicating enhanced eggshell integrity even at low niacin inclusion rates. No significant linear or quadratic trends were detected.

The proportion of shell weight relative to total EW (shell ratio) did not differ among treatments (*P* > 0.05). Shell thickness was significantly affected by dietary treatments (*P* < 0.05). Eggs from quails receiving 10, 20, and 120 mg/kg niacin showed greater shell thickness compared to the control group. The maximum mean value was observed in the 10 mg/kg group (221.83 μm), whereas the control group had the thinnest shells (203.78 μm). However, neither linear nor quadratic effects were statistically significant.

### Egg internal quality traits

As shown in Table 4, dietary niacin significantly affected several internal egg quality parameters. The yolk index did not differ among treatments (*P* > 0.05), although a numerical increase was observed at higher supplementation levels. In contrast, the albumen index increased significantly with dietary niacin (*P* = 0.022), showing a strong linear response (*P* < 0.01); birds receiving 40 mg/kg or more exhibited higher values than the control. Haugh unit scores were also enhanced (*P* < 0.001), rising from 91.47 in the control to 95.15 at 120 mg/kg, representing a 4.0% increase. Overall, the albumen index and Haugh unit increased by 17.9% and 4.0%, respectively, relative to the control (*P* = 0.022 and *P* < 0.001), reflecting a consistent improvement in internal egg quality with increasing niacin level.

**Table 4:** Effect of graded dietary niacin on albumen, yolk, and pigmentation traits in laying quail eggs (*n* = 300)

Parameters	Niacin levels, mg/kg						SEM	Anova	Linear	Quadratic
	0	10	20	40	80	120				
Yolk index	47.90	41.04	44.37	47.70	50.45	50.78	1.524	0.433	0.112	0.659
Albumen index	2.79^b^	2.99^ab^	3.05^ab^	3.12^a^	3.20^a^	3.29^a^	0.046	0.022	0.001	0.227
Haugh unit	91.47^d^	92.42^cd^	93.28^bc^	94.15^ab^	94.59^ab^	95.15^a^	0.291	< 0.001	< 0.001	0.051
L*	61.19	61.48	60.54	59.61	61.38	62.36	0.395	0.484	0.293	0.159
a*	4.82	4.93	4.95	4.97	5.12	4.72	0.092	0.890	0.849	0.259
b*	34.22	36.75	36.59	36.42	37.29	35.68	0.324	0.082	0.452	0.019

^a, b,c,^Means with different superscripts in the same row were significantly different at *P* < 0.05. SEM: standard error means. L*: Lightness; a*: Redness; b*: Yellowness

Regarding yolk colour, no significant differences were found in L* (lightness) or a* (redness) values across treatments (*P* > 0.05). However, b* values (yellowness) showed a significant quadratic trend (*P* < 0.05), with the highest intensity observed at the 80 mg/kg supplementation level.

### Lipid peroxidation and radical scavenging in egg yolk

As shown in Table 5, dietary niacin supplementation had a significant effect on both antioxidant capacity and lipid peroxidation in egg yolk. DPPH radical scavenging activity increased progressively with higher levels of dietary niacin (*P* < 0.001), showing both linear (*P* < 0.001) and quadratic (*P* = 0.030) trends. The lowest activity was recorded in the 10 mg/kg group (6.64%), while the highest value occurred at 120 mg/kg (9.07%), representing a 26.7% increase compared with the control (7.15%).

**Table 5:** Antioxidant status and lipid oxidation markers in egg yolk (*n* = 144) in response to dietary niacin supplementation

Parameters	Niacin levels, mg/kg						SEM	Anova	Linear	Quadratic
	0	10	20	40	80	120				
DPPH (%)	7.154^bc^	6.640^c^	7.101^bc^	7.563^bc^	7.892^b^	9.065^a^	0.1606	< 0.001	< 0.001	0.030
MDA (µmol /kg)	5.018^a^	4.828^a^	4.638^a^	4.447^ab^	3.958^b^	2.258^c^	0.1491	< 0.001	< 0.001	0.016

^a, b,c^Means with different superscripts in the same row were significantly different at *P* < 0.05. SEM: standard error means. DPPH: 2.2 diphenyl-1-picrylhydrazyl. MDA: malondialdehyde. SEM: standard error means

Conversely, lipid peroxidation, expressed by MDA value, decreased markedly with increasing niacin levels (*P* < 0.001), also exhibiting linear (*P* < 0.001) and quadratic (*P* = 0.016) trends. MDA levels declined from 5.02 µmol/kg in the control to 2.26 µmol/kg at 120 mg/kg, corresponding to a 55.0% reduction. Together, these results demonstrate a clear dose-dependent antioxidant response to niacin supplementation in laying quails, characterised by enhanced yolk radical-scavenging capacity and suppressed lipid oxidation.

## Discussion

It is important to note that the basal (control) diet used in this study already contained an analysed niacin concentration of 25.32 mg/kg, slightly above the NRC (1994) recommendation of 20 mg/kg for quails. Therefore, the control group was not niacin-deficient, and the study evaluates supplementation effects above adequate baseline levels. This context likely explains the moderate responses observed at the lower inclusion levels (e.g., 10 mg/kg added, total ≈ 35 mg/kg), as birds were never truly deficient in niacin.

Dietary niacin supplementation produced modest but consistent effects on laying performance, particularly on EP, FCR, and EM. The most favourable responses were associated with moderate supplementation levels (particularly at 40 mg/kg), suggesting a limited dose-responsive effect up to a metabolic threshold beyond which no additional benefits were achieved. This pattern may be explained by niacin’s metabolic role as a precursor of the coenzymes NAD⁺ and NADP⁺, which are critical for cellular respiration, energy metabolism, and nutrient assimilation in poultry (Adebowale et al. 2018; Baghban-Kanani et al. 2019).

Our findings are partially aligned with those of Elsherif et al. (2019), who reported improved performance in laying hens receiving 80 mg/kg of nicotinic acid. Baghban-Kanani et al. (2019) also demonstrated beneficial effects at higher supplementation levels (225–275 mg/kg) in hens.

For instance, Jiang et al. (2011) found no significant improvement in performance parameters of chickens supplemented with niacin up to 120 mg/kg. Such discrepancies may stem from differences in species metabolism (broilers or layers vs. quails), production phase, or basal dietary niacin content. Quails generally exhibit faster metabolic turnover, lower maintenance energy needs, and potentially greater sensitivity to micronutrient modulation (Kılınç et al. 2025), which may explain their response to comparatively lower supplementation levels. In addition to interspecies variation, factors such as the chemical form of niacin (nicotinic acid vs. nicotinamide), feed formulation, and experimental duration may also contribute to these divergent outcomes. Overall, the results suggest that laying quails may reach near-optimal productive responses at lower niacin levels than hens, although the exact requirement remains to be confirmed through broader, multi-phase studies.

Eggshell quality is a key trait that influences both economic value and product integrity, as it determines the likelihood of eggs reaching the consumer intact (Sarmiento-Garcia et al. 2023b). In the current research, the inclusion of 10 mg/kg niacin was associated with a modest improvement in shell strength and thickness, suggesting that shell quality may respond positively to moderate dietary levels of this vitamin. However, no consistent linear or quadratic trend was detected, indicating that this effect was dose-specific rather than progressive across the supplementation range. Such a response may reflect a threshold mechanism, whereby moderate niacin levels are sufficient to support calcium metabolism and shell formation, while further increases confer no additional benefit. Although mineral parameters were not measured in the current experiment, the observed effects may be indirectly related to niacin’s role in calcium and phosphorus metabolism. Niacin contributes to redox reactions involved in energy-dependent mineral transport and matrix formation, acting as a precursor of NAD⁺ and NADP⁺, which participate in osteoblastic activity and calcium deposition (Zhang et al. 2021; Olgun et al. 2022). Adebowale et al. (2019) also reported improved dietary mineral retention and utilisation efficiency in birds supplemented with niacin, which could enhance calcium availability for shell formation. Previous studies have shown similar responses in other avian species. El-Husseiny et al. (2008) reported increased shell thickness and shell ratio at 150 mg/kg of niacin, while Baghban-Kanani et al. (2019) and Gungor et al. (2003) noted similar tendencies at higher inclusion levels (175–1500 mg/kg). In contrast, Elsherif et al. (2019) found no significant response in hens supplemented with 20–80 mg/kg. The comparatively lower effective level observed in quails may reflect species-specific differences in calcium metabolism, nutrient partitioning, or niacin utilisation.

In the current study, dietary niacin supplementation produced a moderate linear increase in albumen index and Haugh unit, suggesting a trend toward improved internal egg quality in laying quails. These responses may be related to enhanced albumen stability and protein integrity, potentially associated with the metabolic functions of niacin as a precursor of NAD and NADP coenzymes involved in energy metabolism and antioxidant defence (Obianwuna et al. 2022). The concurrent increase in yolk antioxidant capacity (higher DPPH activity and lower TBARS values) is consistent with the hypothesis that improved oxidative protection helps maintain albumen structure and functionality. In comparison with previous studies in laying hens, the effects observed here were moderate but more consistent at lower dietary levels. For instance, Gungor et al. (2003) reported enhanced albumen index and Haugh unit at very high supplementation levels (1500 mg/kg), but not at lower doses (250–1000 mg/kg). In contrast, studies by Baghban-Kanani et al. (2019) and Olorunsola et al. (2021) found no significant effects of niacin supplementation (30–275 mg/kg) on Haugh unit in hens. Elsherif et al. (2019) similarly reported no improvements in the Haugh unit and even observed a reduction in albumen index with 20 mg/kg supplementation. Such discrepancies may reflect species-specific metabolic efficiency, differences in basal niacin content, or variations in dietary formulation. Quails, with their faster metabolism and lower maintenance energy requirements (Sarmiento-García et al. 2023a), could exhibit a more pronounced response to moderate niacin supplementation compared with larger avian species.

Regarding yolk colour, niacin supplementation produced a quadratic trend in b values, peaking at 80 mg/kg, suggesting a moderate increase in yolk yellowness, an attribute positively valued by consumers (Başer et al. 2025). This supports earlier findings in hens by Gungor et al. (2003), who found higher yolk pigmentation at 500–1500 mg/kg. This result aligns with findings in laying hens by Gungor et al. (2003), who found higher yolk pigmentation at higher supplementation levels (500–1500 mg/kg niacin). The underlying mechanism may involve changes in lipid metabolism and lipoprotein synthesis, which could indirectly affect carotenoid transport and deposition in the yolk. Niacin has been reported to influence hepatic lipid metabolism and nutrient absorption in poultry (Zhang et al. 2021), which may partly explain the observed increase in yolk pigmentation. Interestingly, this response contrasts with results reported in poultry meat, where Jiang et al. (2011) and Zhang et al. (2021) observed reduced colour intensity with niacin supplementation. Such discrepancies may reflect tissue-specific differences in pigment accumulation and oxidative metabolism. While yolk colour depends mainly on carotenoid uptake and deposition (Kojima et al. 2022; Sarmiento-Garcia et al. 2023b), muscle colour in poultry depends on myoglobin concentration, oxidative stability, and muscle fibre characteristics (Weng et al. 2022). These processes are influenced by metabolic pathways in which niacin acts as a cofactor, modulating mitochondrial and antioxidant activity (Akinyemi and Adewole 2021). Overall, the results are consistent with a modulatory role of niacin in yolk pigmentation, potentially linked to improved nutrient utilisation and lipid-soluble pigment transport in laying quails.

Niacin plays a crucial role in maintaining redox homeostasis through its function as a precursor of the NAD⁺/NADH and NADP⁺/NADPH coenzymes, which participate in key metabolic pathways such as glycolysis, the tricarboxylic acid (TCA) cycle, and β-oxidation. These coenzymes act as hydrogen carriers and support the regeneration of antioxidant systems, including glutathione and thioredoxin (Zhang et al. 2021; Olgun et al. 2022). Moreover, NADPH availability modulates oxidative stress through its role as a substrate for NADPH oxidase and as a cofactor for antioxidant enzyme systems (Tarafdar and Pula 2018). In this study, dietary niacin supplementation was associated with higher yolk antioxidant values, possibly reflecting its central role in redox metabolism. Yolk DPPH values increased linearly with dietary niacin level, reaching the highest mean at 120 mg/kg, whereas MDA concentrations declined quadratically and were lowest at the same inclusion rate. These results suggest that niacin-derived cofactors enhance cellular reducing capacity and suppress lipid peroxidation in the yolk. The observed trends are consistent with Keskin and Karul (2022), who reported increased serum antioxidant capacity with niacin supplementation in rats.

However, not all research concurs: Baghban-Kanani et al. (2019) found no significant changes in serum MDA, superoxide dismutase, overall antioxidant capacity, or glutathione at niacin doses (175, 225 and 275 mg/kg), which may relate to matrix-specific sensitivity. The yolk lipid fraction, rich in polyunsaturated fatty acids, is particularly prone to oxidative damage and therefore more responsive to shifts in redox balance than serum. Niacin’s influence on NAD(P)H availability could exert stronger protective effects in such lipid-rich environments by stabilising peroxyl radicals and enhancing the recycling of lipid-soluble antioxidants such as tocopherols. This may explain why yolk-based assays detect changes more readily than systemic biomarkers. Nevertheless, future research should include systemic antioxidant indicators (e.g., serum or hepatic markers) to better integrate yolk responses with whole-body oxidative status.

Together, these results support a “dual requirement” interpretation, in which moderate niacin supplementation (~ 40 mg/kg) appears sufficient to optimise productive traits. In contrast, higher inclusion (up to 120 mg/kg) is required to reinforce antioxidant protection. This divergence likely reflects the differential partitioning of niacin-derived cofactors between metabolic and redox functions. At lower levels, the NAD⁺ and NADP⁺ pools primarily support energy metabolism and nutrient utilisation, while additional NADPH availability at higher levels enhances antioxidant regeneration.

From a practical standpoint, the optimal dietary niacin level may therefore depend on production objectives. Systems focused on feed efficiency and short storage cycles could benefit from moderate supplementation (~ 40 mg/kg), whereas those targeting improved oxidative stability or shelf-life might consider higher inclusion levels. Nevertheless, the economic and practical value of such adjustments remains to be quantified, and further research is warranted to determine whether enhanced antioxidant capacity translates into measurable improvements in egg quality retention under commercial storage conditions.

## Conclusions

In summary, dietary niacin supplementation enhanced productive performance, egg quality, and yolk antioxidant capacity in laying quails. The optimal level for performance traits was approximately 40 mg/kg, whereas higher inclusion (up to 120 mg/kg) was required to maximise yolk oxidative stability. These results suggest that niacin exerts a dual role in supporting productivity and antioxidant defence.

Although similar dose–response effects have been reported in laying hens, the present study provides species-specific evidence indicating that the optimal range for each trait may differ, reflecting variations in metabolic sensitivity and nutrient utilisation among poultry species. Overall, niacin supplementation influenced quail performance and antioxidant status in a dose-dependent, non-linear manner.

Nevertheless, the study’s exploratory nature and limited scale warrant further long-term research before any modification to official niacin recommendations can be proposed.

## Data Availability

The authors declare that all data and materials used in this study comply with field standards and are available upon request.

## Declarations

### Generative AI and AI-assisted technologies in the writing process

During the preparation of this work, the authors used ChatGPT (OpenAI) to improve language clarity and to assist in rephrasing sections of the manuscript. After using this tool, the authors carefully reviewed, edited, and verified all content to ensure accuracy and integrity. The authors take full responsibility for the content of the publication.
